# Use of a medication-based algorithm to identify advanced Parkinson's disease in administrative claims data: Associations with claims-based indicators of disease severity

**DOI:** 10.1016/j.prdoa.2020.100046

**Published:** 2020-02-26

**Authors:** Nabila Dahodwala, Amy R. Pettit, Jordan Jahnke, Pengxiang Li, Vrushabh P. Ladage, Prasanna L. Kandukuri, Jorge Zamudio, Yash J. Jalundhwala, Jalpa A. Doshi

**Affiliations:** a Department of Neurology, Perelman School of Medicine, University of Pennsylvania, 330 South 9th Street, 2nd Floor, Philadelphia, PA 19107, USA; b Leonard Davis Institute of Health Economics, University of Pennsylvania, Philadelphia, PA, USA; c Center for Public Health Initiatives, University of Pennsylvania, Philadelphia, PA, USA; d Department of General Internal Medicine, Perelman School of Medicine, University of Pennsylvania, 1223 Blockley Hall, Philadelphia, PA 19104, USA; e AbbVie Inc., 1 N Waukegan Road, North Chicago, IL 60064, USA

**Keywords:** Parkinson's disease, Disease staging, Clinical indicators, Claims data, Medicare

## Abstract

**Introduction:**

Lack of a gold standard definition for advanced Parkinson's Disease (APD), coupled with absence of disease severity information in diagnostic codes, hinders use of large administrative databases for conducting population health and comparative effectiveness studies.

**Methods:**

Using pharmacy claims data, we created an algorithm to identify APD: any 30-day average levodopa equivalent dose (LED) >1000 mg/day. Using 2013 100% U.S. Medicare claims, we applied this algorithm and used multivariate logistic regression to examine associations between assigned APD status and claims-based indicators of PD severity (any deep brain stimulation, fall, hallucinations, walker, wheelchair, specialty bed, dementia diagnosis, skilled nursing facility, hospice), adjusting for sociodemographic, clinical, and treatment characteristics. Levodopa >1000 mg/day, levodopa >800 mg/day and LED >800 mg/day were used in sensitivity analysis.

**Results:**

In our sample (*N* = 144,703), 20% were assigned APD status based on the LED >1000 mg/day cut-off. This group had significantly higher odds of having each claims-based indicator, compared with those assigned mild-moderate PD status. Odds ratios were highest for indicators for any DBS (OR: 2.96; 95% CI:2.75–3.19) and specialty bed (OR:2.15, 95% CI: 1.99–2.32) and lowest for fall (OR:1.27; 95% CI:1.20–1.34) and dementia diagnosis (OR:1.21; 95% CI:1.18–1.25). Results based on alternative approaches were similar.

**Conclusions:**

Medicare patients classified as having APD via a pharmacy claims-based algorithm had higher odds of having claims-based clinical markers of APD, compared with patients categorized as having mild-moderate PD. This proxy strategy could facilitate future claims-based studies and warrants further refinement and validation using medical records or other clinical sources.

## Highlights

• Prescription claims can be used to calculate average daily levodopa-equivalent dose.
• An algorithm (LED >1000 mg/day) was used to identify advanced Parkinson's in claims.
• 20% of people with Parkinson's disease in a Medicare sample aged 65+ met this definition.
• The remaining 80% of the sample was classified as having mild/moderate disease.
• The advanced group had higher odds of having clinical markers of advanced disease.

## 1 Introduction

Parkinson's disease (PD) is the second most prevalent age-related neurodegenerative disorder after Alzheimer's disease and is associated with motor symptoms (e.g., tremor) and non-motor symptoms (e.g., dementia) [1,2]. Advancing disease is associated with increasing morbidity, fluctuations in symptom control, impairment in activities of daily living, and mortality [3]. As with other progressive conditions, disease staging is useful for treatment planning and disease management and also facilitates research aimed at understanding the burden of disease, disease progression, clinical heterogeneity, and treatment and adherence patterns. Administrative claims databases are a valuable resource for conducting such population-level studies because they offer an economical and efficient way to study large groups of patients treated in real-world settings [4]. For example, a large claims-based study could compare real-world outcomes associated with two different treatments for individuals with APD. The ability to identify patients according to severity is also a critical tool for insurers who seek to utilize administrative claims data to target patients for disease management or medication therapy management programs.

In the United States, a particularly rich research resource is administrative claims data from Medicare, a federally funded health insurance program that provides coverage to 98% of Americans who are 65 years of age and older as well as those who are younger but meet certain disability criteria [5,6]. Medicare data are particularly valuable to identify patients by PD severity, given that PD most often afflicts the elderly and typically advances in severity with age, and the Medicare Part D Medication Therapy Management Program may offer an opportunity to better manage APD patients. Yet the ability to conduct claims-based research focused on PD severity is hindered by the fact that current *International Classification of Diseases (ICD)* diagnosis codes – a typical strategy for identifying patients belonging to a specific diagnostic group – do not incorporate PD stage. There is also no standard clinical algorithm for identifying PD stage in administrative claims datasets, which limits their utility for studies of individuals with advanced disease.

Efforts to develop an algorithm for identifying advanced Parkinson's disease (APD) in claims data are complicated by several factors. First, there is no gold standard clinical definition of APD on which to base such an algorithm. Second, initial efforts to define disease progression focused primarily on balance changes as documented by a standard rating scale (the Hoehn and Yahr, or H&Y scale) [7], but such clinical ratings are not available in claims data. Third, clinician approaches to documenting the presence of complications associated with advanced disease, such as dementia, may lead to variable sensitivity and specificity when they are used as a claims-based proxy. On the one hand, a clinician who is concerned about a patient's cognitive functioning may include a diagnostic code for dementia in the absence of a full assessment of whether the individual meets criteria for a formal diagnosis. On the other hand, a patient who does qualify for a dementia diagnosis but does not disclose cognitive issues during a clinical visit may subsequently remain undiagnosed, or a provider may note symptoms in a medical chart but not include the diagnostic code in insurance claims.

Despite these challenges, several studies have attempted to identify advanced disease via claims-based proxy identifiers. A study of the economic costs of PD by Johnson and colleagues [8] distinguished newly diagnosed individuals from those with more advanced disease based on evidence of new claims for an ambulatory assistive device (i.e., walker or wheelchair; proxy for H&Y stage 4). Subsequent studies also captured a subgroup defined by admission to a skilled nursing facility [9] or long-term care facility (proxy for H&Y stage 5) [10]. While informative, using medical services to proxy APD may have more limited utility in studies requiring clinical precision. For example, while ambulatory assistive devices are more commonly used in APD as compared to earlier in the disease process, they are not universally needed. Thus, such proxies may fail to identify a large proportion of patients in the early stages of APD in claims data, representing a missed opportunity to examine real-world treatment patterns in those most likely to benefit from targeted management of APD. At the same time, the use of ambulatory assistive devices in an individual with PD may be related to other age-related comorbidities rather than PD itself, which could lead to some earlier stage patients being misclassified as having APD.

We sought to advance efforts to detect APD in claims data in two key ways, based on the emerging global consensus that a broader array of clinical indicators is important for identifying APD [3]. First, we built an algorithm using pharmacy claims only, given the easier accessibility to prescription claims (as opposed to medical claims) data for many key stakeholders in the U.S., including stand-alone Medicare Part D plans, prescription benefit managers, and pharmacies. Second, in addition to refining and testing the medication-based algorithm, we also sought to assess–for the first time–whether disease group assignment based on the algorithm was associated with other established clinical markers of PD severity.

## 2 Methods

### 2.1 Data source and sample

Our retrospective study utilized 2013 100% Medicare claims from the Chronic Condition Data Warehouse, which includes all individuals in the U.S. who are covered by fee-for-service Medicare. The dataset included medical claims from Medicare Part A (hospital, skilled nursing facility, limited home health services, and hospice) and Part B (outpatient hospital, physician, physician-administered drugs, other outpatient, and durable medical equipment); Medicare Part D prescription claims (outpatient prescription drug event) files; and a personal summary file with the beneficiary's demographic and enrollment information.

Our analytic sample consisted of beneficiaries aged 65 or older who had at least one inpatient or outpatient claim with a diagnosis code of PD (ICD-9-CM code 332.0). To be included in our study (Supplementary Fig. 1), individuals had to be continuously enrolled in fee-for-service Medicare Parts A and B as well as a stand-alone Part D (prescription drug) plan and alive throughout the study period (i.e., the calendar year 2013). Individuals were also required to have received levodopa treatment (at least one levodopa medication claim in 2013, with or without additional treatments) and to have complete data available for all key covariates. This approach to case definition criteria has been shown to optimize both sensitivity and specificity when using administrative claims to identify individuals with PD [11,12].

### 2.2 Conceptual framework for identifying APD via medication-based proxy

As noted earlier, there is no single standard clinical definition of APD or diagnostic test to identify it. Nonetheless, levodopa is often considered the gold standard medication therapy for symptomatic treatment of PD [13], and changes in its use correspond to typical changes in symptoms as the disease advances. While levodopa improves mobility and decreases disability, the progressive neurodegeneration that occurs with advancing PD is associated with the need for higher levodopa doses to reduce symptomatic “off” time. The medical literature, including reports of medication dosing in clinical studies of patients with advanced disease, supports the idea that higher doses of levodopa are a marker of increasing disability and serve as a proxy for clinical features of advanced disease such as motor complications and dyskinesias (Supplementary Table 1). Whereas more frequent levodopa dosing has also been identified as a marker of advanced disease [3], it is difficult to confidently discern this level of detail from prescription claims data.

Given that patients could have been taking additional medications for their PD, we calculated levodopa equivalent doses (LEDs) in addition to levodopa dosing. We chose dosing thresholds based on a review of dosing reported in clinical trials of advanced therapies such as deep brain stimulation (DBS), which are indicated in APD when symptoms have progressed to motor fluctuations and increasing “off” time. Details on the individual studies included in our dosing review may be found in Supplementary Table 1. We calculated LEDs based on a previously published algorithm from a systematic review that considered all anti-PD oral medications [14]. Our dosing threshold for APD was any 30-day average LED >1000 mg/day. To categorize patients, we calculated the daily medication dosing for each individual for each day in 2013 and looked for any 30-day period during the study year when the average dosing met the threshold. Patients with dosing satisfying this criterion were categorized as having APD. We assigned APD status to patients who were on consistently higher doses (e.g., 30-day average > 1000 mg/day throughout the study year) and also those who may have received a higher dose for a shorter period of time (e.g., one month or more). We opted for this less stringent criterion based on the reasoning that higher doses–regardless of whether they were well-tolerated and continued–were likely to signal advanced disease. We categorized all PD patients in the final sample as having either APD or mild-moderate PD (≤1000 mg/day).

### 2.3 PD disease severity indicators

Next, in keeping with prior studies [8,9] and recently published consensus guidelines aimed at identifying clinical indicators of the transition to APD [3], we identified multiple clinical indicators for advanced PD that are available in administrative claims. These included presence of: 1) any medical claim reflecting a Current Procedural Terminology (CPT) code indicating current or recent DBS treatment (e.g., device placement, programming); 2) any medical claim reflecting an ICD-9 code for a fall; 3) selected diagnostic codes for hallucinations; 4) a durable medical equipment (Part B) claim for a walker, a proxy for patients at H&Y stage 4; 5) a durable medical equipment claim for a wheelchair, also a proxy for patients at H&Y stage 4; 6) a durable medical equipment claim for a specialty bed, a proxy for patients at H&Y stage 5; 7) a medical claim containing a diagnostic code for dementia (any subtype); 8) a medical claim for care in a skilled nursing facility, indicated when inpatient rehabilitation is needed due to a decline in function; or 9) a medical claim for hospice care, indicated at end of life. Because our intention was to test our algorithm by examining generally contemporaneous markers of APD rather than to predict clinical outcomes, we captured claims for these indicators at any point during the study year.

### 2.4 Covariates

The covariates of interest included patient sociodemographic characteristics (age, sex, race, region); prescription drug hierarchical condition categories (RxHCC) risk score [15]; and whether patients had an outpatient claim for a neurologist visit during the study year (yes/no). The RxHCC score is a risk-adjustment score created using a series of medical condition categories coded from patients' medical claims and has been used to adjust for comorbidities and/or potential selection biases in prior studies among Medicare patients. A higher RxHCC score indicates a higher comorbidity burden. We captured neurologist care as a covariate based on prior evidence that patients who see a neurologist are more likely to be on PD medications and may experience different rates of relevant outcomes [16,17].

### 2.5 Statistical analysis

For descriptive analyses, chi-square tests were used for categorical variables and *t*-tests were used for continuous variables. We employed logistic regressions to examine relationships between assigned APD status (as the main independent or predictor variable; reference value was mild-moderate PD) and the clinical indicators of advanced disease described above (as the dependent or outcome variables), while adjusting for relevant covariates. We hypothesized that patients classified by the medication-based algorithm as having APD would have greater odds of having known clinical indicators of greater disease severity.

Primary analyses utilized the LED >1000 mg/day threshold for identifying APD. In sensitivity analyses, we used three alternative methods of assigning APD status: 1) any 30-day average levodopa dose >1000 mg/day; 2) any 30-day average levodopa >800 mg/day and; 3) any 30-day average LED >800 mg/day). We selected the 800 mg threshold to test in sensitivity analyses because it is a less conservative cut-off for APD that was still within the range of medication dosing for APD found in our literature review. The University of Pennsylvania Institutional Review Board deemed the study exempt from informed consent procedures because no data were collected directly from patients.

## 3 Results

Table 1 shows sociodemographic, clinical, and treatment characteristics for our final sample of 144,703 individuals as well as group characteristics using the LED >1000 mg/day version of our algorithm. A total of 28,974 (20%) people were categorized as having APD, with the remaining 80% classified as mild-moderate PD. We found statistically significant differences across all characteristics we examined. The APD group had a lower proportion of the oldest patients (>80 years old) as compared to the mild-moderate group (34.3% vs. 49.3%, *P* < 0.001), and also a higher proportion of men (58.0% vs. 46.1%, *P* < 0.001). A greater proportion of those in the APD group had a claim for a neurologist visit during the study year (84.2% vs. 66.3%, *P* < 0.001), as compared to the mild-moderate PD group.

**Table 1:** Sample characteristics, overall and by assigned PD severity (LED >1000 mg/day algorithm)^a^, ^b^.

Characteristic	Overall (*N* = 144,703)		Mild-Moderate PD (*n* = 115,729)		Advanced PD (*n* = 28,974)	
	N	Percent	N	Percent	N	Percent
Age, years						
65–69	16,702	11.5%	11,964	10.3%	4738	16.4%
70–74	27,882	19.3%	20,685	17.9%	7197	24.8%
75–79	33,141	22.9%	26,039	22.5%	7102	24.5%
≥80	66,978	46.3%	57,041	49.3%	9937	34.3%
Sex						
Male	70,127	48.5%	53,321	46.1%	16,806	58.0%
Race						
White	126,830	87.6%	100,878	87.2%	25,952	89.6%
Black	6666	4.6%	5860	5.1%	806	2.8%
Other	11,207	7.7%	8991	7.8%	2216	7.6%
Region						
Northeast	28,430	19.6%	23,061	19.9%	5369	18.5%
Midwest	38,687	26.7%	30,487	26.3%	8200	28.3%
South	52,885	36.5%	43,040	37.2%	9845	34.0%
West	24,701	17.1%	19,141	16.5%	5560	19.2%
RxHCC score, mean (SD)^c^	1.48 (0.46)		1.50 (0.46)		1.40 (0.45)	
Neurologist visit^d^	101,146	69.9%	76,762	66.3%	24,384	84.2%

PD, Parkinson's disease; RxHCC score, prescription drug hierarchical condition category risk score.

a Patients were classified based on an algorithm derived from prescription claims. Patients with any 30-day average levodopa equivalent dose (LED) >1000 mg/day were classified as advanced; all others were assigned mild-moderate status.

b Comparisons of patient subgroups for all variables listed were statistically significant at the *P* < 0.001 level, using chi-square tests for categorical variables and a t-test for the continuous variable.

c RxHCC scores in the overall sample ranged from 0.72 to 6.30; scores in the mild/moderate PD group ranged from 0.72 to 6.30 and scores in the APD group ranged from 0.72 to 5.33.

d Indicates patient had an outpatient claim for a neurologist visit during the study year.

**Fig. 1:**
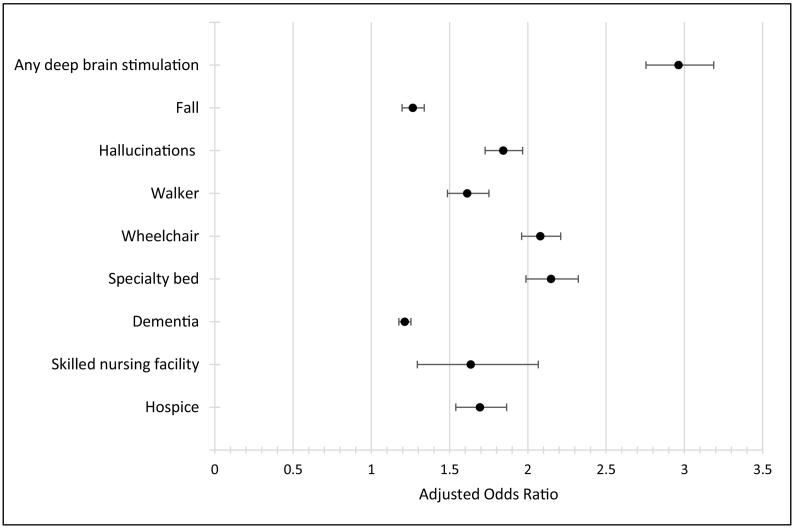
Associations between assigned APD status and claims-based clinical indicators of advanced disease^a,b^. APD, Advanced Parkinson's Disease. ^a^Patients were classified as having advanced disease based on an algorithm derived from prescription claims (any 30-day average levodopa equivalent dose [LED] >1000 mg/day). Patients classified as mild-moderate (via 30-day average LED ≤1000 mg/day) were the reference group. Logistic regressions adjusted for sociodemographic characteristics (age, sex, race, region), clinical characteristics (RxHCC score), and treatment characteristics (any outpatient visit with a neurologist during study year). Error bars represent 95% confidence intervals. ^b^Any deep brain stimulation was defined as the presence of any CPT code indicating current or recent treatment (e.g., device placement or programming).

As shown in Table 2, each of the disease severity indicators we examined were more prevalent among the APD group, with the exception of dementia, which was more common in the mild-moderate group (39.2% vs. 33.6%, *P* < 0.001). After controlling for relevant covariates, however, the APD group had significantly higher odds of having a claim for each of our clinical indicators of disease severity (see Fig. 1). Increased odds were highest for claims related to any DBS (OR: 2.96; 95% CI: 2.75–3.19) and a specialty bed (OR: 2.15, 95% CI: 1.99–2.32) and lowest for dementia (OR: 1.21; 95% CI: 1.18–1.25) and falls (OR: 1.27; 95% CI: 1.20–1.34).

**Table 2:** Prevalence of clinical indicators of advanced Parkinson's disease, by assigned disease severity group^a^, ^b^.

Indicator	Overall (N = 144,703)		Mild-Moderate PD (n = 115,729)		Advanced PD (n = 28,974)	
	N	Percent	N	Percent	N	Percent
Any deep brain stimulation^c^	3690	2.6%	1880	1.6%	1810	6.2%
Fall	8099	5.6%	6298	5.4%	1801	6.2%
Hallucinations	4724	3.3%	3246	2.8%	1478	5.1%
Walker	7907	5.5%	6250	5.4%	1657	5.7%
Wheelchair	12,784	8.8%	9739	8.4%	3045	10.5%
Specialty bed	7711	5.3%	5974	5.2%	1737	6.0%
Dementia	55,085	38.1%	45,347	39.2%	9738	33.6%
Skilled nursing facility	374	0.3%	267	0.2%	107	0.4%
Hospice	2545	1.8%	1913	1.7%	632	2.2%

PD, Parkinson's disease.

a Patients were classified based on an algorithm derived from prescription claims. Patients with any 30-day average levodopa equivalent dose (LED) >1000 mg/day were classified as advanced; all others were classified as mild-moderate.

b Comparisons of patient subgroups for all variables listed were statistically significant at the *P* < 0.001 level.

c Deep brain stimulation was defined as the presence of any CPT code indicating current or recent treatment (e.g., device placement or programming).

This pattern of findings held across each dosing threshold we examined (Table 3). Classification based on levodopa dosing alone resulted in a slightly lower rate of APD (17%; data not shown), whereas application of the lower dose cutoffs resulted in a slightly higher percentage of patients being classified as APD (27% for levodopa >800 mg/day and 30% for LED >800 mg/day; data not shown).

**Table 3:** Associations between alternate methods of assigning APD status and clinical indicators of advanced disease^a^.

	Levodopa dose >1000 mg/day (*n* = 24,851)	Levodopa dose >800 mg/day (*n* = 39,631)	LED dose >800 mg/day (*n* = 43,790)
APD indicators	OR (95% CI)	OR (95% CI)	OR (95% CI)
Any deep brain stimulation^b^	2.96 (2.74–3.19)	2.47 (2.30–2.66)	2.41 (2.24–2.59)
Fall	1.24 (1.18–1.30)	1.23 (1.17–1.30)	1.26 (1.19–1.33)
Hallucinations	1.71 (1.61–1.82)	1.63 (1.53–1.73)	1.74 (1.63–1.86)
Walker	1.58 (1.47–1.71)	1.54 (1.43–1.67)	1.61 (1.47–1.75)
Wheelchair	2.02 (1.91–2.13)	1.93 (1.83–2.04)	2.00 (1.89–2.13)
Specialty bed	2.11 (1.96–2.27)	2.04 (1.89–2.19)	2.09 (1.93–2.27)
Dementia	1.18 (1.15–1.22)	1.20 (1.16–1.23)	1.21 (1.17–1.25)
Skilled nursing facility	1.72 (1.39–2.12)	1.62 (1.30–2.01)	1.44 (1.12–1.85)
Hospice	1.71 (1.57–1.86)	1.67 (1.53–1.82)	1.69 (1.53–1.86)

APD, Advanced Parkinson's disease; CI, confidence interval; LED, levodopa equivalent dose; OR, odds ratio.

a Dosage cutoffs represent various approaches to classifying patients as having APD, based on an algorithm derived from prescription claims. Patients meeting the dose criterion indicated were classified as having advanced disease. Logistic regressions adjusted for sociodemographic characteristics (age, sex, race, region), clinical characteristics (RxHCC score), and treatment characteristics (any outpatient visit with a neurologist during study year). The reference group is those who did not meet the dosage criterion (i.e., those classified as mild-moderate status). All comparisons were significant at the *P* < 0.001 level, with the exception of skilled nursing facility for the levodopa dose >800 mg group (*P* < 0.005).

b Deep brain stimulation was defined as the presence of any CPT code indicating current or recent treatment (e.g., device placement or programming).

## 4 Discussion

This study builds on prior approaches to identifying individuals with advanced Parkinson's disease (APD) in administrative claims databases [[8], [9], [10],^18^]. Using comprehensive Medicare claims data from 2013 for individuals aged 65 or older, we tested a claims-based algorithm based on medication dosing calculated from prescription drug claims, using several dosage thresholds. For the first time in the published literature, we examined whether the disease status assigned via our algorithm was associated with a variety of claims-based clinical indicators of disease severity. Using a medication threshold of any 30-day average levodopa equivalent dose (LED) >1000 mg/day over the course of the 1-year study period, we found that approximately 20% of our sample of Medicare patients with PD were classified as having advanced disease, with the remaining 80% classified as having mild-moderate disease. The group identified as having APD had significantly higher odds of each indicator of disease severity that we examined, including having a claim for any DBS placement or treatment, one or more falls, hallucinations, a walker, a wheelchair, a specialty bed, a diagnosis of dementia, use of a skilled nursing facility, and hospice care.

It is difficult to compare our results to existing APD prevalence statistics, as findings from population-based studies of PD have varied widely due to differences in patient sampling and methodology. Among studies using H&Y stages to define disease severity, for example, prevalence of stage 3 disease has ranged from 6%–25%, stage 4 from 15%–35%, and stage 5 from 4%–10% [[19], [20], [21], [22], [23]]. In prior studies that utilized an APD definition related to degree of disability, the proportion of cases categorized as severe (4%–27%) was similar to our findings (i.e., 20% classified as APD) [[19], [20], [21], [22], [23]]. The rates detected by alternate versions of our algorithm ranged from 17% (with levodopa dosing >1000 mg/day) to 30% (with a lower dosing threshold, LED >800 mg/day).

Our approach to identifying APD using claims data is conceptually very similar to one used in a contemporaneous study by Weir and colleagues, who used LED >1100 mg/day as one of several proxy markers of APD in a study of economic costs in the United Kingdom [18]. Our findings offer insights into the potential limitations and benefits of using an algorithm based solely on medication dosage. Contrary to expectations for a progressive disease, our APD group had a lower percentage of older patients, which likely reflects the fact that our algorithm would not detect older patients with advanced disease who cannot tolerate higher doses of dopaminergic therapy. Similarly, we also observed a slightly lower mean RxHCC score in our APD group and a narrower range of scores compared with the mild/moderate group. This could mean that our dose-based algorithm may have misclassified some medically complex APD patients who were unable to tolerate higher doses of PD medications, yet it could also reflect the fact that individuals with more comorbid conditions may be less likely to survive during the advanced stages of PD. In addition, although our APD group had higher odds of having a dementia diagnosis as compared to the mild-moderate group, the difference between groups was smaller than might be expected. This could reflect variation in coding practices for dementia and also likely reflects the fact that patients with dementia are at increased risk for psychosis and thus may receive lower doses of levodopa [24]. Additional clinical factors may impede the tolerability of higher doses of dopaminergic therapy, such as the presence of orthostatic hypotension or dyskinesias, leading to misclassification of some APD patients as mild/moderate. An algorithm based solely on medication dose over the course of a single year may lack sensitivity in these cases (i.e., miss certain cases of APD), despite the fact that we would expect it to have high specificity (i.e., a low rate of false positives for APD). One exception might be individuals with mild/moderate disease who are tremor-dominant and medication resistant, which could lead to doses exceeding the 1000 mg/day limit and misclassification as APD. At the same time, our medication-based algorithm may have been more sensitive than other approaches in identifying patients with advanced disease who had a lower level of physical disability. For example, our APD group had a lower rate of falls (6.2%) than the rate found in a cohort of individuals with APD defined by an incident claim for an ambulatory-assistive device (31.9%) [9]. An additional strength of our approach is the ability to identify individuals with APD in databases outside the U.S. that contain pharmacy information.

Our findings should be interpreted in the context of several caveats. We examined claims data from 2013, before the U.S. Food and Drug Administration had approved additional APD treatments (i.e., continuous infusion treatments), and it would be important to include claims for these therapies in future studies examining markers of advanced disease. Our LED-based algorithm will be able to accommodate newer dopamine-based therapies or those that are developed in the future, but if alternative, non-dopamine therapies prove to be effective in the management of PD, this algorithm would need to be adapted. Further, whereas frequency of dosing (i.e., ≥5 doses per day) has now been identified as a clinical indicator of APD [3], we were not able to derive precise frequency of dosing from prescription drug claims, which do not have the clinical detail that is available in electronic medical records. Prescription claims also reflect filled prescriptions, and actual medication use may vary [3]. Our focus on a single year of data also means our findings on ambulatory assistive devices and specialty beds would reflect only those individuals who obtained a new piece of equipment during the study year. We would not capture individuals who may have been using devices obtained prior to the study period or those who may have been using assistive devices they obtained via other means, such as a loan program through a local senior center. As a result, our findings may underestimate the true prevalence of walker, wheelchair, and specialty bed use. In addition, our sample did not include individuals under 65 or those covered by Medicare Advantage plans, which provide Medicare Parts A, B, and D coverage together and were not included in our fee-for-service claims data source. Whereas claims data lack the richness of detail available through medical record review, studies using claims data allow for an efficient, relatively inexpensive, and faster way to study patterns of disease management across large samples.

We chose our algorithm threshold to maximize specificity at the expense of sensitivity, to ensure that the sample designated as advanced was most likely to have advanced disease. However, the current algorithm can be adapted based on the specific research question. For example, a study focused on detecting APD cases with dementia might opt to use a lower LED threshold to identify cases, or to add supplementary criteria such as requiring an additional medical claim for dementia or a pharmacy claim for dementia medication. Some studies might also benefit from examination of medication use over a longer period of time (e.g., 2 years), to enable detection of APD cases where an initial increase in dosage was later lowered due to clinical considerations.

Our findings represent an important step forward in the effort to identify and test a valid claims-based algorithm to identify patients with APD, which will facilitate population-level research such as comparative effectiveness, health care utilization, and economic burden studies. Future studies are needed to validate the algorithm in other datasets, against medical record data, and to further explore its sensitivity and specificity in identifying individuals with APD.

The following are the supplementary data related to this article.

Supplementary Table 1 Levodopa dosing in clinical trials of patients with advanced Parkinson’s disease Supplementary Table 1

Supplementary Fig. 1 Sample selection diagram. CCW: Chronic Condition Data Warehouse; ICD-9-CM: *International Classification of Diseases, 9*^*th*^*Revision, Clinical Modification;* PD: Parkinson’s disease. Supplementary Fig. 1

## Funding statement

This study was supported and funded by 10.13039/100006483AbbVie Inc. AbbVie participated in study design; analysis and interpretation of data; writing, reviewing, and approving the manuscript; and the decision to submit the article for publication. All authors contributed to the development of the manuscript and maintained control over the final content.

*Declaration of Interest:* ND receives grant funding from AbbVie (research grant), Roche (clinical trial site investigator), Eli Lilly (clinical trial site investigator), Cala Health (clinical trial site investigator) and Medtronic (training grant). PL has no disclosures related to the submitted work; outside of the submitted work, PL has consultancy relationships with HealthStatistics LLC, Avalon Health Economics LLC, and Robert Ohsfeldt LLC. PLK, JZ, and YJJ are employees of AbbVie and may own stock/shares in AbbVie Inc. JAD reports serving as an advisory board member or consultant for Allergan, Ironwood Pharmaceuticals, Janssen, Kite Pharma, Merck, Otsuka, Regeneron, Sarepta, Sage Therapeutics, Sanofi, Shire, and Vertex; and has received research funding from AbbVie, Biogen, Humana, Janssen, Novartis, PhRMA, Regeneron, Sanofi, and Valeant (to support her and/or coauthors Drs. Pettit and Li, Mr. Jahnke, and Mr. Ladage). All are unrelated to the submitted work. Her spouse holds stock in Merck and Pfizer. JJ, ARP, and VPL have no additional disclosures to report.

## CRediT authorship contribution statement

**Nabila Dahodwala:**Conceptualization, Methodology, Formal analysis, Writing - original draft, Supervision, Project administration, Funding acquisition.**Amy R. Pettit:**Writing - review & editing, Visualization.**Jordan Jahnke:**Methodology, Software, Formal analysis, Data curation, Writing - review & editing.**Pengxiang Li:**Methodology, Software, Formal analysis, Writing - review & editing.**Vrushabh P. Ladage:**Writing - review & editing, Project administration.**Prasanna L. Kandukuri:**Writing - review & editing.**Jorge Zamudio:**Writing - review & editing.**Yash J. Jalundhwala:**Conceptualization, Resources, Writing - review & editing.**Jalpa A. Doshi:**Conceptualization, Methodology, Formal analysis, Resources, Writing - review & editing, Supervision, Funding acquisition.

## References

[1] Dorsey E.R., Constantinescu R., Thompson J.P., Biglan K.M., Holloway R.G., Kieburtz K. (2007). Projected number of people with Parkinson disease in the most populous nations, 2005 through 2030. Neurology.

[2] Marras C., Beck J.C., Bower J.H., Roberts E., Ritz B., Ross G.W. (2018). Prevalence of Parkinson’s disease across North America. NPJ Parkinsons Dis..

[3] Antonini A., Stoessl A.J., Kleinman L.S., Skalicky A.M., Marshall T.S., Sail K.R. (2018). Developing consensus among movement disorder specialists on clinical indicators for identification and management of advanced Parkinson’s disease: a multi-country Delphi-panel approach. Curr. Med. Res. Opin..

[4] Huse D.M., Schulman K., Orsini L., Castelli-Haley J., Kennedy S., Lenhart G. (2005). Burden of illness in Parkinson’s disease. Mov. Disord..

[5] National Center for Health Statistics (2014). Health, United States, 2013: With Special Featureon Prescription Drugs.

[6] Kaiser Family Foundation (2019). An Overview of Medicare. http://files.kff.org/attachment/issue-brief-an-overview-of-medicare.

[7] Goetz C.G., Poewe W., Rascol O., Sampaio C., Stebbins G.T., Counsell C. (2004). Movement Disorder Society Task Force report on the Hoehn and Yahr staging scale: status and recommendations. Mov. Disord..

[8] Johnson S., Davis M., Kaltenboeck A., Birnbaum H., Grubb E., Tarrants M. (2011). Early retirement and income loss in patients with early and advanced Parkinson’s disease. Appl. Health Econ. Health Policy.

[9] Kaltenboeck A., Johnson S.J., Davis M.R., Birnbaum H.G., Carroll C.A., Tarrants M.L. (2012). Direct costs and survival of Medicare beneficiaries with early and advanced Parkinson’s disease. Parkinsonism Relat. Disord..

[10] Johnson S.J., Kaltenboeck A., Diener M., Birnbaum H.G., Grubb E., Castelli-Haley J. (2013). Costs of Parkinson’s disease in a privately insured population. Pharmacoeconomics.

[11] Jain S., Himali J., Beiser A., Ton T.G., Kelly-Hayes M., Biggs M.L. (2015). Validation of secondary data sources to identify Parkinson disease against clinical diagnostic criteria. Am. J. Epidemiol..

[12] Noyes K., Liu H., Holloway R., Dick A.W. (2007). Accuracy of Medicare claims data in identifying Parkinsonism cases: comparison with the Medicare Current Beneficiary Survey. Mov. Disord..

[13] Agid Y., Ahlskog E., Albanese A., Calne D., Chase T., De Yebenes J. (1999). Levodopa in the treatment of Parkinson’s disease: a consensus meeting. Mov. Disord..

[14] Tomlinson C.L., Stowe R., Patel S., Rick C., Gray R., Clarke C.E. (2010). Systematic review of levodopa dose equivalency reporting in Parkinson’s disease. Mov. Disord..

[15] Robst J., Levy J.M., Ingber M.J. (2007). Diagnosis-based risk adjustment for Medicare prescription drug plan payments. Health Care Financ. Rev..

[16] Willis A.W., Schootman M., Evanoff B.A., Perlmutter J.S., Racette B.A. (2011). Neurologist care in Parkinson disease: a utilization, outcomes, and survival study. Neurology.

[17] Dahodwala N., Willis A.W., Li P., Doshi J.A. (2016). Prevalence and correlates of anti-Parkinson drug use in a nationally representative sample. Mov. Disord. Clin. Pract..

[18] Weir S., Samnaliev M., Kuo T.C., Tierney T.S., Walleser Autiero S., Taylor R.S. (2018). Short- and long-term cost and utilization of health care resources in Parkinson’s disease in the UK. Mov. Disord..

[19] Mutch W.J., Dingwall-Fordyce I., Downie A.W., Paterson J.G., Roy S.K. (1986). Parkinson’s disease in a Scottish city. Br. Med. J. (Clin. Res. Ed.).

[20] Wermuth L., Pakkenberg H., Jeune B. (2002). High age-adjusted prevalence of Parkinson’s disease among Inuits in Greenland. Neurology.

[21] Wermuth L., von Weitzel-Mudersbach P., Jeune B. (2000). A two-fold difference in the age-adjusted prevalences of Parkinson’s disease between the island of Als and the Faroe Islands. Eur. J. Neurol..

[22] Wermuth L., Joensen P., Bunger N., Jeune B. (1997). High prevalence of Parkinson’s disease in the Faroe Islands. Neurology.

[23] Chio A., Magnani C., Schiffer D. (1998). Prevalence of Parkinson’s disease in northwestern Italy: comparison of tracer methodology and clinical ascertainment of cases. Mov. Disord..

[24] Connolly B.S., Lang A.E. (2014). Pharmacological treatment of Parkinson disease: a review. JAMA.

