# Supplementary material for: Use of a medication-based algorithm to identify advanced Parkinson's disease in administrative claims data: Associations with claims-based indicators of disease severity
Source: Clin Park Relat Disord. 2020 Feb 26;3:100046. doi: 10.1016/j.prdoa.2020.100046 (PMC8298763; doi:10.1016/j.prdoa.2020.100046)
Supplement: Supplementary Table 1 — Levodopa dosing in clinical trials of patients with advanced Parkinson’s disease [file mmc1.docx]

Supplementary Table 1: Levodopa dosing in clinical trials of patients with advanced Parkinson’s disease

| **First Author (Year)** | **Type of Study** | **Clinical Definition of Advanced PD** | **Levodopa Equivalent Dose^a^ Mean (SD) mg/day** |
| --- | --- | --- | --- |
| Lang (2016) | Review of safety data from 4 clinical trials of levodopa-carbidopa intestinal gel | Motor complications | 1080.7 (65.3) |
| Odekerken (2016) | Randomized clinical trial comparing motor, cognitive, and behavioral outcomes 3 years after DBS to the GPi or STN | Motor complications, dyskinesias | 1060 (range 657-1860) GPI-DBS; 605(range 411-875) STN-DBS |
| Martinez-Martin (2015) | Open-label, prospective study comparing apomorphine infusion to intrajejunal levodopa infusion in APD | H&Y, UPDRS | 2017.1 (857.2) IJLI; 5 1,059.3 (231.5 ) apomorphine |
| Fernandez (2015) | Open-label, prospective study of levodopa-carbidopa intestinal gel in APD | Motor complications | 1082.9 (582.1) |
| Olanow (2014) | Double-blind, double-dummy, randomized study of continuous intrajejunal infusion of levodopa-carbidopa intestinal gel in APD | Motor complications | 1005.4 (373.6) levodopa intestinal gel; 1123.5 (477.9) Levodopa immediate release |
| Schuepbach (2013) | Randomized controlled trial of neurostimulation vs best medical therapy for Parkinson’s disease with early motor complications | Motor complications, dyskinesias | 918.8 (412.5) Neurostimulation; 966.9 (416.5) medical therapy |
| Amara (2012) | Prospective case series of the effects of unilateral subthalamic nucleus DBS on sleep quality in APD | UPDRS | 1198.4 (69.52) |
| Antonini (2011) | Prospective study comparing DBS to continuous subcutaneous apomorphine infusion in APD | H&Y stage ≥3, motor complications, dyskinesias, UPDRS | 785.4 (251.9) CSAI; 770 (440.9) STN-DBS |
| Weaver (2009) | Randomized controlled trial comparing bilateral DBS to best medical therapy in APD | H&Y, motor complications, dyskinesias | 1289 (546) best medical therapy; 1281 (521) DBS |
| Nyholm (2008) | Retrospective study of patients with APD who received enteral levodopa/carbidopa infusion | Motor complications | 1014 (480) [oral levodopa] |
| Slowinski (2007) | Case series of unilateral DBS of the subthalamic nucleus in APD | Motor complications, dyskinesias | 978 (395) |
| Deuschl (2006) | Randomized-pairs trial comparing DBS to best medical therapy in APD | UPDRS, dyskinesias | 1176 (517) neurostimulation; 1175 (461) medical therapy |
| Wider (2006) | Case series in patients with APD treated with DBS to the subthalamic nucleus | Dyskinesias | 1138 (507) |
| Visser-Vandewalle (2005) | Case series in patients with APD treated with bilateral subthalamic nucleus stimulation | Motor complications, dyskinesias | 1133 (383) |
| Stocchi (2005) | Open-label study of continuous intestinal levodopa administration in patients with APD | Dyskinesias | 1233.3 (98.3) |
| Loher (2002) | Case series of DBS to globus pallidus in patients with APD | Motor complications, dyskinesias, H&Y | 1126.7 unilateral DBS; 1235.5 bilateral DBS^b^ |
| Simuni (2002) | Case series of bilateral stimulation of the subthalamic nucleus in patients with APD | Motor complications, UPDRS | 1946 (338) |

APD, advanced Parkinson’s disease; LED, levodopa equivalent dosing; DBS, deep brain stimulation; H&Y, Hoehn & Yahr scale; IJLI, intrajejunal levodopa gel infusion; UPDRS, Unified Parkinson Disease Rating Scale; CSAI, continuous subcutaneous infusion of apomorphine

^a^Each study calculated and reported LED dosing.

^b^Standard deviation was not reported.
