# Supplementary material for: Use of a medication-based algorithm to identify advanced Parkinson's disease in administrative claims data: Associations with claims-based indicators of disease severity
Source: Clin Park Relat Disord. 2020 Feb 26;3:100046. doi: 10.1016/j.prdoa.2020.100046 (PMC8298763; doi:10.1016/j.prdoa.2020.100046)
Supplement: Supplementary Fig. 1 — Sample selection diagram. CCW: Chronic Condition Data Warehouse; ICD-9-CM: International Classification of Diseases, 9thRevision, Clinical Modification; PD: Parkinson’s disease. [file mmc2.docx]

Supplementary Figure 1. Sample selection diagram

Excluded for missing data on

key covariates

(n=3,847)

2013 CCW Medicare PD claim (ICD-9-CM: 332.0) with age ≥65 years

(N=497,660)

Final Sample

(N=144,703)

Continuous Medicare Parts A and B fee-for-service coverage throughout 2013 (n=412,009)

Continuous Medicare Part D coverage throughout 2013

(n=234,692)

Alive throughout 2013

(n=231,275)

Any claim for an oral PD medication (n=161,278)

≥1 medication claim for levodopa, alone or in combination with

other treatment

(n=148,550)

Excluded for not having >1 medication claim for levodopa, alone or in combination with

other treatment

(n=12,728)

Excluded for not having a claim for an oral PD medication

(n=69,997)

Excluded for not having continuous Medicare Part D coverage throughout 2013

(n=177,317)

Excluded due to death prior to

end of coverage year 2013

(n=3,417)

Excluded for not having continuous Medicare Parts A and B fee-for-service coverage throughout 2013 (n=85,651)

CCW: Chronic Condition Data Warehouse; ICD-9-CM: *International Classification of Diseases, 9^th^ Revision, Clinical Modification;* PD: Parkinson’s disease.
